# The micromammals

**DOI:** 10.1093/g3journal/jkae073

**Published:** 2024-06-05

**Authors:** Susan L Forsburg

**Affiliations:** Program in Molecular and Computational Biology, University of Southern California, Los Angeles, CA 90089-2910, USA

## Abstract

In this editorial, Senior Editor Susan Forsburg examines the reasons to keep studying eukaryotic microbes like *S. pombe* and *S. cerevisiae*—and other yeasts, algae, amoeba, and fungi—even as genetic and genomic technologies now allow manipulation and study of practically any organism. She explores the challenges and opportunities of working in these tiny organisms, pointing to the substantial biology their study has uncovered.

Years ago, I attended a seminar by John Carbon, who played a key early role in developing molecular genetics tools in the budding yeast *Saccharomyces cerevisiae* and in identifying the centromere. He began by telling a story. Carbon suggested that if a Martian scientist arrived on Earth unaware of the internal combustion engine, it would be difficult to dissect its function if you started by examining a Mercedes.

“But,” he said, “if our Martian was observant, he would realize that the lawnmower in the corner of the garage works by the same principle as the Mercedes, while much simpler in design. For example, the Mercedes may have more sparkplugs, and they may look a little different, but the basic mechanism is the same.” Carbon went on to say if humans are the Mercedes, then yeast is the lawnmower.

I have often over the years used this effective analogy of the lawnmower and the Mercedes to explain the utility of simple single-celled eukaryotes in understanding fundamental principles of human biology. But is there still a place to study the lawnmower today?

Scroll through recent issues of *G3*, and you will see the answer is an emphatic YES! Of course, *S. cerevisiae* was the first eukaryotic genome sequenced and remains a major developmental platform for a variety of sophisticated genomic techniques. From analysis of multigene traits to metabolomics to synthetic biology to modeling human chromosome dynamics, we are still learning a lot from the humble budding yeast (Robinson *et al.* 2023; Garge *et al.* 2024; McNamara *et al.* 2024; Papp *et al.* 2024). And it's not alone. Our choice of lawnmowers continues to expand in important ways, which leads to another story.

Thirty years ago, when I first started my lab, I was one of just a few fission yeast geneticists in the United States of America (most *Schizosaccharomyces pombe* people were in Europe or Japan). I frequently got asked what I hoped to learn from studying another yeast. It's worth reminding you, dear reader, that “yeast” as a term is more general than “animal.” It describes a wide variety of fungi, and it is estimated that fission and budding yeast diverged 350 MY ago (Hoffman *et al.* 2015). For example, studying both was essential to uncover the essential role of CDK kinases in both G1-S (*S. cerevisiae*) and G2-M (*S. pombe*) cell cycle transitions. Each has maintained fundamental processes that the other has lost, from typical histone-H3K9 methylated heterochromatin (*S. pombe*) to sophisticated peroxisomes (*S. cerevisiae*) (e.g. Forsburg 1999; Hoffman *et al.* 2015). Our understanding of fundamental cell biology has been enriched by having both of these organisms available, allowing a compare-and-contrast approach.

And it's not just these 2 yeasts. Our perusal of the *G3* table of contents will show us a variety of other model eukaryotic microbes including other yeasts (e.g. *Candida)*, filamentous fungi (e.g. *Aspergillus*), algae (e.g. *Chlamydomonas*), and amoeba (e.g. *Dictyostelium*) that continue to give us important and useful insights. Significantly, investigators from mammalian systems have often picked up microbes to study their pathways of interest, leading to the nickname of “micromammals.” But these eukaryotic microbes are not only valuable as tools to understand our own human biology. Some of them are pathogens of plants or animals, providing agricultural or medical utility in uncovering their cellular mysteries, and some of them are “just” exquisite additions to the tapestry of life on earth (Kang *et al.* 2022; Kurbessoian *et al.* 2023; Sun *et al.* 2023; Welgemoed *et al.* 2023).

I tasked my stellar team of associate editors to help me identify key challenges and opportunities in the eukaryotic microbe world and provide examples of the kinds of *G3* studies that help address these challenges.

A first area might be described as the “low hanging fruit”: the parsing of gene function in already established systems. There is still much to learn about how these common organisms function. Well-done studies on single genes that advance the field always have a home in *G3*, and we’ve also seen targeted high-throughput approaches to comprehensively identify new functions or pathways involving known factors (Lao *et al.* 2018; Chatfield-Reed *et al.* 2022; Lin *et al.* 2022; Li *et al.* 2024).

An area that invites further study is identifying gene function of unknown genes, including but not limited to those with human orthologs. It's been reported that the vast majority of publications on human gene function actually ignore a large number of genes, the “unknowns.” Many of these have homologs in at least some model systems (Wood *et al.* 2019). This area is ripe for systemic analysis and comprehensive gene-by-gene studies.

Identification of new organisms and comparison to existing genomes is another area for which *G3* provides a significant venue. Several editors highlighted the robust and comprehensive genome description of a new species *Schizosaccharomyces osmophilus* (Jia *et al.* 2023). Significant value was added by extensive cross-species genomic comparisons that uncover evolutionary trajectories for various chromosome elements and the mitochondria. *G3* is interested in novel genomes that provide important insights into a range of microbial life. Cross-species comparisons have been particularly helpful in leveraging these new assemblies (Celia-Sanchez *et al.* 2022; Librais *et al.* 2024; van Westerhoven *et al.* 2024).

It's key to move past what's known and familiar, and ask, what is the new, unexpected, or simply cool biology that may exist in these systems? For example, *S. osmophilus* requires a high-osmolarity environment to grow, suggesting there will be interesting and potentially novel pathways to uncover. Importantly, we shouldn’t limit ourselves to genes found in humans, and there's much to learn from fungal-specific or even species-specific genes. It's worth remembering that transformative discoveries like CRISPR often come from basic studies of simple systems (reviewed in Doudna and Charpentier 2014). Consistent with the long history in technology development in fungi, we see CRISPR applications here too (Sizova *et al.* 2021; Gervais *et al.* 2023).

A neglected area of new biology is differentiation. Many of our standard model microbes undergo sexual differentiation, of course, and yeast mating and meiosis have been widely studied for many years, with ongoing insights (Trainor *et al.* 2021). But there is also the potential in some species for multicellular interactions or differentiation involved in host–pathogen engagement. These problems may be accessible through developing omic approaches, as well as leveraging classical and molecular genetics. Importantly, many of these are not the usual model systems, highlighting our interest in “cool biology.” Some examples of papers in these areas include rather unusual fungi with predatory habits (Abdullah and Borts 2001; Chen *et al.* 2022; Lohmar *et al.* 2022; Benson *et al.* 2024). This again highlights our broad interest in biology generally, not limited to human applications.

As we review these papers, it is apparent that the authors have genuine enthusiasm for their tiny subjects, and we sense their evident delight as a new secret is uncovered. Additionally, these are examples of substantial biology, not small increments. *G3* continues to offer a unique home for useful and well-done studies that provide insights into broad aspects of microbial biology. Who knew that there were so many secrets to find in a lawnmower!
